# Notes and News

**Published:** 1887-07-02

**Authors:** 


### NOTES AND NEWS.

COLLECTED AND COMMUNICATED.

MR. PIETRO J. MICHELLI has been appointed Secretary of the Dreadnought Seamen's Hospital, Greenwich. Mr. Michelli has shown by his work at St. Mary's that he is a skilled administrator, and able to enforce successfully the best principles of hospital administration. We congratulate the Greenwich Hospital authorities upon their selection, and are sure that the old Dreadnought will soon take a leading position amongst the metropolitan medical charities because of its increased efficiency.

MR. THOMAS RYAN has been appointed Secretary of St. Mary's Hospital, and has been selected out of some hundreds of candidates by seventeen votes to two. Mr. Ryan's work at Queen Charlotte's Hospital abundantly justifies the appointment, and the Governors of St. Mary's Hospital are to be congratulated upon the wise selection they have made with practical unanimity in choosing a successor to Mr Michelli.

WE understand that Mr. G. Owen Ryan, late steward of the Dreadnought Seamen's Hospital, has been appointed Secretary to the Queen Charlotte's Lying-in Hospital, *vice* Mr. Thomas Ryan, resigned.

THE Gordon Hospital for Fistula, etc., has just received a grant of twenty-five guineas from the Mercers' Company.

THE competition for the Romford Cottage Hospital has resulted in the selection of Mr. J. S. Cordor and Mr. Frank Brown as joint architects. Forty designs were sent in.

THE sum of £37 17*s.* has been sent to the building fund of the Great Northern Central Hospital as the proceeds of entertainments given by the Theseus Dramatic Club.

THE town of Nairn, in the north of Scotland, spends an extraordinarily small sum annually upon its hospital. But though this sum is so very small it seems to be too large for the population to raise. The total income of the institution during the past year was £169 10*s.* 5*d.*, whilst the expenditure was £250. The greater world would have a better opinion of the town and county of Nairn if they would give more adequate support to their hospital.

ON Monday evening a well-attended meeting of the working-men in Leigh, Lancashire, was held in the Drill Hall, to consider the propriety of appointing a working-men's committee to act in conjunction with the general committee for the purpose of establishing an infirmary for Leigh and the surrounding districts. We are glad to see that the working-men are taking so active a part in this good work.

THE Stanley Hospital at Liverpool is not in a very flourishing financial condition. It is situated in a locality where its help is much needed; it is officered by an excellent staff; it is highly appreciated by the public and those who make use of its privileges; but still a considerable sum is required not only to meet its current expenses, but also to develop its usefulness. The people of the surrounding district should feel bound to free it from debt, and to give it all the resources required for greater efficiency.

THE working-men of Tynemouth and the Friendly and Temperance Societies of the town and neighbourhood are coming forward energetically in support of the local infirmary. This is an occasion of great satisfaction to the clergy and other principal inhabitants of the town and district. If the working-classes can only be roused to a due sense of their responsibility in these matters there is a brighter future for hospitals.

THE new Victoria Jubilee buildings at the Donnybrook Hospital, the first stone of which was laid on the 29th ult. by H.R.H. Prince Albert Victor, are to be on the pavilion system, and will include special accommodation for consumptive patients and those suffering from other diseases of the chest. The wards will have a south and south-westerly aspect, and will be sheltered on the north and east. Every modern improvement which can in any way ameliorate the condition of the invalids will be adopted. The rooms are to be heated by hot-water-pipes, in addition to the open fireplaces, and thus an even temperature will be maintained both day and night, combined with ample ventilation. This hospital, it is said, will be most favourably situated for the treatment of consumptive patients. The architect for the new buildings is Mr. Rawson Carrolls, F.R.I.B.A.

The following vacancies are announced this week, the amount of the salary being placed in each case after the name of the institution :—

**Honorary Medical Officers.**

University College. Assistant Physician, Assistant Surgeon, and Assistant Obstetric Physician to the Hospital.

**Resident Medical Officers.**

Ipswich Hospital. House Surgeon. Doubly-qualified.—£100.
Kent County Asylum. Second Assistant. Registered.—£120.
Wakefield Asylum. Clinical Assistant. No salary.

**Non-resident Medical Officers.**

Birmingham, Mason College. Professor of Physiology.
Wandsworth Union. Dispenser. Qualified.—£80.

**Matrons and Superintendents.**

Bristol General Hospital. Locum Tenens for Night Superintendent.
Matron and Resident Midwife for a Maternity Hospital.—£40. Apply to Geo. Fox, 53, Princes Street, Manchester.

**Trained Nurses.**

Bradford Eye and Ear Hospital. Two.—£25.
Bristol Infirmary. Assistant and Night.
Carlisle Infirmary. One.—£24.
Chelsea Hospital for Women. Night.—£20.
Norfolk and Norwich Hospital. Two.—£24.

---

Dr. James Tuxford, of Boston, has received from Mr. Harrison, of Messrs. Marshall and Snellgrove, a communication to the effect that members of that firm have contributed a sum of £50 to the Boston Cottage Hospital. Mr. Harrison himself, who is a native of Boston, and Mrs. Harrison give a further £50, making in all £100 towards the extension fund.

---

The *Catholic Times* announces its readiness to receive mounted photographs and albums for distribution to the provincial hospitals. At the present moment the *Times* has a large stock of albums and unmounted photographs, a dozen of which photographs, mounted ready for framing, it will send to any hospital upon application.

---

Some time ago the working-men of Leeds showed themselves anxious to do much more for the local hospital than had been their habit in former times. With this view an extensive organisation was created, and has apparently begun to produce good fruits. A number of collections have been made at different factories and other places of business, amounting in the aggregate to £72 11*s.* 6*d.* If so much can be raised in a few weeks by the workmen of a few firms, what may not be looked for when the whole of the operatives of Leeds and its neighbourhood put their hands to the work?

---

The Royal Albert Edward Infirmary at Wigan has held its annual meeting. The work done during the year has been of the usual amount and character. It is very satisfactory to note that this hospital is not only able to meet its expenses, but to put aside something out of its income for a rainy day. The expenditure last year was £4,777 9*s.* 4*d.*, whilst the income was £5,129 12*s.* 6*d.*, and, as *The Wigan Observer* says, "What can be more satisfactory? The continued increase of the workmen's contributions is a special reason for congratulation, the total sum paid over to the institution last year from the weekly contributions and Hospital Saturday movements being no less than £2,759 16*s.*" Can any other town in Great Britain of a similar size show such generosity on the part of its working people?

---

The London Temperance Hospital possesses 120 beds, only 60 of which are at present in use, in consequence of a debt of £6,500, which encumbers the managers. If this debt were paid off the whole of the beds might speedily be brought into requisition.

---

A few days ago a fire broke out at the East-end Children's Hospital, and much excitement and alarm were created. By some unaccountable accident the lift had caught fire on the second floor, but the outbreak was subdued before the flames had time to spread.

---

At the Princess Alice Hospital, Eastbourne, all patients who were able, together with the nurses and servants, assembled at ten o'clock in the large male ward for Divine service, at which special Jubilee hymns were sung. This over, a large portrait of her Majesty was drawn for and wcn by a male patient, who intends having it framed, and then presenting it to the hospital to be hung in the ward in which he was placed. This event was followed by a distribution of knives to the male, silver thimbles to the female patients and servants, and silver brooches or studs to the nurses. Dinner was served at mid-day, and then all who were able were driven to Miss Thompson's, 2, The Goffs, from whose windows a capital view was obtained of the festivities in South Fields. Tea and supper were both served here and the heartiest thanks were expressed to the kind entertainer. At eight o'clock the party was driven back to the hospital, which had been prettily illuminated and decorated. The royal portraits presented to the hospital by her Majesty were hung with wreaths of flowers made by the patients and nurses. From the garden of the hospital the beacon fires on Beachy Head and Willingdon Hill could be seen.

---

The Bishop of Bedford's statement at the Shoreditch Town Hall, that hospitals would in all probability come upon the State unless they were more adequately supported by voluntary contributions, has excited no little apprehension throughout the country. The provincial press unites with the metropolitan in deprecating any such catastrophe. It is felt that the honour of England demands that the hospitals, which up to the present time have been maintained solely by the generosity of the charitable, should not only continue to be so supported, but, as the wealth of the country increases, they and similar institutions should expand with the progress of the times.

---

At Hull, the Royal Infirmary is not receiving so large a share of public help as it ought to receive, and it imperatively requires. Whether it is that the means of the people are smaller, or that the funds are diverted into other channels, is not very apparent. It seems to us that in Hull, as in other towns, hospitals have a decided priority of claim above almost every other institution. Yet the amount annually contributed by the public towards them, as compared with the amounts contributed for some other purposes, is ridiculously small. It may be hoped that the day is not far distant when hospitals will take their proper place in public estimation as the first of all the charities.

At the Stockton-on-Tees Hospital a tea and entertainment to the patients were given on Thursday, June 23, in celebration of the Queen's Jubilee.

The following arrived too late for publication in last week's issue:—"We are given to understand, but I can scarcely credit the report, that the Governing Body of St. Bartholomew's Hospital have forbidden any sisters or nurses to leave the building on the 21st June. May we presume to inquire the reason of so arbitrary an order? Is it impossible for the work of the institution to be carried on satisfactorily unless the entire nursing staff is confined within its walls? Doubtless the authorities have some excellent reason for this apparently extraordinary decision, but we cannot refrain from asking why those who labour for the public good should be absolutely debarred from any share in the public rejoicing?—An Indignant Observer."

At the Queen Victoria Convalescent Home for sick children, Belfast, an enthusiastic meeting of the committee was held in the hospital. A vote of congratulation to her Majesty the Queen upon attaining the jubilee of her reign was proposed by Mr. Frame, seconded by Mr. Curry, supported by Mrs. McKanse and Mr. Canning, and passed with acclamation. Subscriptions amounting to upwards of £40 were handed in, and the meeting was concluded by the singing of the National Anthem.

The Duke of Portland presided at a festival dinner in aid of the West-end Hospital for Paralysis and Diseases of the Nervous System, situated at 73, Welbeck Street, which was held recently in the Whitehall rooms of the Hôtel Métropole. The institution was founded in 1878. During the meeting Mr. F. Edgecombe Dunsford announced that the subscriptions amounted to £330. A portion of the band of the Coldstream Guards, under the conductorship of Mr. C. Thomas, performed during dinner, afterwards a programme of music was rendered, the singing of the National Anthem closing the proceedings.

A demonstration and church parade by members of the United Friendly and Temperance Societies was held on Sunday last in aid of the Children's Hospital, Great Ormond Street. The starting-points were the Chester Coffee Tavern, South Highgate, Highbury Station Road, Yorkshire Stingo Bars, and Argyle Square. A special service was held at St. James's Church, Queen's Square, where a sermon was preached by the Rev. Dacre Craven. Collections made on the road and at the church amounted to over £80.

The sum of £6,500 has been divided among 15 local medical charities at Birmingham as the net available proceeds of the recent Hospital Saturday collection. The total amount collected was £6,820 6*s.* 6*d.* as against £6,703 9*s.* 3*d.* last year. Thus Hospital Saturday at Birmingham realises more than half as much as the same collection in London, although London is eight times bigger than Birmingham, and probably eighteen times wealthier. At the meeting of the sub-committee the principles on which the distribution of the fund should be made were considered at some length. It was finally decided to adhere to the plan hitherto followed, viz., that the amount to each charity should be proportioned to the ordinary current expenditure, as stated in the last audited report.

Mr. Henry Irving has set an excellent example to other theatrical managers in sending 50 guineas to the Hospital Sunday Fund, in lieu of the collection-box that was placed in the vestibule of the Lyceum last year As, however, there are few London managers who can afford the substantial aid which Mr. Irving has given would it not be a graceful act on the part of the patrons of the different theatres to assist managers with their contributions? A small coin from each occupant of a box or a stall during the week would provide a handsome sum in the course of a twelvemonth.

It will interest our readers to know that, with a view to preserving in the memories of their hearers the impressions of Hospital Sunday, copies of the *Sunday Fund Special Number* of The Hospital were distributed at the doors of many churches and chapels after the services. Among the rest may be mentioned St. Michael's, Chester Square (Canon Fleming's), 500 copies; St. Mary's, Newington (Rev. Mr. Palmer's), 500; All Saints', Kensington (Canon Trench), 500; Metropolitan Tabernacle (Mr. Spurgeon's), 1,000; City Temple (Rev. Dr. Parker's), 500. The proprietors of The Hospital presented to the Committee of the Saturday Fund 10,000 copies of the first special number, which were sold at the stalls in aid of the Fund, and four thousand five hundred copies were sent by post to ministers of religion of all denominations.

The late Bishop of Sodor and Man, who was a sincere friend of hospitals, had promised to take part in an open-air service at Douglas Head on the 31st July, in aid of the Isle of Man General Hospital. Although the Bishop is now no more, it will not be forgotten that one of the last contemplated public acts of his life was of such a benevolent and truly Christian character. The annual collection at the Isle of Man hotels and boarding-houses will take place on Sunday, July 31st, and the church collections on Sunday, August 7th.

The annual meeting of the Governors of the Dundee Royal Infirmary has just been held in the Guildhall under the presidency of Mr. James Luke. The secretary read the report, which showed that the work done during the year had been in excess of that of the previous year, and that the funds of the institution had slightly increased. The directors recorded their satisfaction with the good order and management which prevailed in all the departments of the infirmary, and the efficient manner in which Superintendent Dr. M'Cosh and the matron, Miss Finlyson, had discharged their duties. We are glad to see the managers of infirmaries recognising and making public their appreciation of the value of the services rendered by permanent officials.

