# Norfolk and Norwich Hospital

**Published:** 1887-07-09

**Authors:** W. Earnshaw

**Affiliations:** Secretary


### NORFOLK AND NORWICH HOSPITAL.

### A Correction.

*To the Editor of* The Hospital.

Sir,—In The Hospital of 2nd of July, page 233, "Trained Nurses, Norfolk and Norwich Hospital, Two—£24," should be "Norfolk and Norwich Trained Nurses' Home," an establishment entirely unconnected with this Hospital. The applications, in consequence of the above error, are being constantly received by the Lady Superintendent here, who asks to have the mistake corrected.—Yours faithfully,

W. Earnshaw, *Secretary*.

